# Changes in incidence of HPV-related cancers in South Africa (2011–21): a cross-sectional analysis of the South African National Cancer Registry

**DOI:** 10.1016/S2214-109X(25)00065-8

**Published:** 2025-06

**Authors:** Jaimie Z Shing, Sizeka Mashele, Adino T Tsegaye, Bianca Da Costa Dias, Eric A Engels, Admire Chikandiwa, Meredith S Shiels, Judith Mwansa-Kambafwile, Erica S Stephens, Carole Metekoua, Danping Liu, Loretto J Carvajal, Aimée R Kreimer, Mazvita Muchengeti

**Affiliations:** Division of Cancer Epidemiology and Genetics, National Cancer Institute, Rockville, MD, USA; National Cancer Registry, National Health Laboratory Service, Johannesburg, South Africa, Swiss Tropical and Public Health Institute, Allschwil, Switzerland, University of Basel, Basel, Switzerland; Division of Cancer Epidemiology and Genetics, National Cancer Institute, Rockville, MD, USA; Centre for HIV and STIs, National Institute for Communicable Diseases, National Health Laboratory Service, Johannesburg, South Africa; Division of Cancer Epidemiology and Genetics, National Cancer Institute, Rockville, MD, USA; Department of Obstetrics and Gynaecology, Infectious Disease and Oncology Research Institute, University of the Witwatersrand, Johannesburg, South Africa; Division of Cancer Epidemiology and Genetics, National Cancer Institute, Rockville, MD, USA; National Cancer Registry, National Health Laboratory Service, Johannesburg, South Africa, School of Public Health, School of Public Health, University of Cape Town, Cape Town, South Africa; Division of Cancer Epidemiology and Genetics, National Cancer Institute, Rockville, MD, USA; National Cancer Registry, National Health Laboratory Service, Johannesburg, South Africa; Division of Cancer Epidemiology and Genetics, National Cancer Institute, Rockville, MD, USA; Division of Cancer Epidemiology and Genetics, National Cancer Institute, Rockville, MD, USA, Agencia Costarricense de Investigaciones Biomédicas, Fundación INCIENSA, San José, Costa Rica; Division of Cancer Epidemiology and Genetics, National Cancer Institute, Rockville, MD, USA; National Cancer Registry, National Health Laboratory Service, Johannesburg, South Africa

## Abstract

**Background:**

Understanding human papillomavirus (HPV)-related cancer epidemiology in South Africa is crucial for informing cancer prevention in this high-burden country. We aimed to describe HPV-related cancer incidence in South Africa between 2011 and 2021.

**Methods:**

For this cross-sectional study, we obtained data on cancer incidence from the South African National Cancer Registry and population estimates from Statistics South Africa. We calculated age-standardised incidence per 100 000 person-years for cervical carcinoma, and vulvar, vaginal, penile, oropharyngeal, and anal squamous cell carcinoma among people aged 15 years and older by sex, year, age, and race. Average annual percentage changes (AAPCs) were calculated using the Joinpoint Regression Program.

**Findings:**

Between Jan 1, 2011 and Dec 31, 2021, the overall cervical carcinoma incidence was 30·4 cases per 100 000 person-years (95% CI 30·2 to 30·6), which was highest in females aged 55–64 years (58·5 cases per 100 000 person-years [57·5 to 59·5]); incidence was stable between 2011 and 2016 and began to decline in 2016 (AAPC −2·7% [95% CI −10·8 to −0·2]). The incidence of vulvar squamous cell carcinoma (2·3 cases per 100 000 person-years [2·2 to 2·4]), vaginal squamous cell carcinoma (0·7 cases per 100 000 person-years [0·7 to 0·7]), and female-anal squamous cell carcinoma (0·6 cases per 100 000 person-years [0·6 to 0·7]) increased between 2011 and 2021 (AAPC 8·0% [95% CI 5·3 to 13·9] for vulvar squamous cell carcinoma; 3·2% [0·5 to 6·6] for vaginal squamous cell carcinoma; and 8·5% [2·0 to 23·2] for anal squamous cell carcinoma). The largest increase in vulvar squamous cell carcinoma between 2011 and 2021 was observed among females aged 15–44 years (AAPC 10·0% [8·2 to 13·4]) and 45–54 years (AAPC 10·7% [8·2 to 14·1]). The incidence of penile squamous cell carcinoma (1·4 cases per 10 000 person-years [1·3 to 1·4]) and anal squamous cell carcinoma in males (0·4 cases per 100 000 person-years [0·4 to 0·5]) increased between 2011 and 2021 (AAPC 6·9% [3·8 to 10·7] for penile squamous cell carcinoma; 9·3% [6·7 to 12·7] for anal squamous cell carcinoma). For both sexes, oropharyngeal squamous cell carcinoma trends were stable. The incidence of cervical carcinoma, vulvar squamous cell carcinoma, and vaginal squamous cell carcinoma was highest among Black females; penile squamous cell carcinoma was highest among Black males; anal squamous cell carcinoma in males was similar by race; and oropharyngeal squamous cell carcinoma was highest among White and Coloured individuals.

**Interpretation:**

The incidence of non-cervical anogenital cancers is rapidly increasing in South Africa. The incidence of most HPV-related cancers is high among Black individuals, especially for cervical and vulvar cancers, potentially due to disproportionately high HPV–HIV co-infection prevalence among young Black females. HIV prevention and continued HPV vaccination efforts are crucial for reducing HPV-driven cancers in the future.

**Funding:**

South African National Health Laboratory Services and US National Cancer Institute Intramural Research Program.

## Introduction

The prevalence of human papillomavirus (HPV) infection varies by world region.^1^ The highest burden of cervical HPV infections is in sub-Saharan Africa,^1^ where HIV infection prevalence is also the highest globally.^2^ In South Africa, cervical HPV prevalence among females with normal cytology ranges from 18 to 75% depending on the province, age group, and HIV co-infection status,^3^ and in 2022, according to UNAIDS, 7·6 million South Africans were living with HIV, of whom 4·8 million were females aged 15 years and older. Among people with HIV, HPV infection clearance rates are reduced and the risk of persistent HPV infection is elevated compared with people without HIV.^4^ This is concerning because persistent HPV infection is a major risk factor for developing cervical, vulvar, vaginal, penile, anal, and oropharyngeal cancers.^5^

In 2022, cervical cancer was the second most common cancer and the leading cause of cancer-related mortality among females in South Africa.^6^ Understanding the epidemiology of HPV-related cancers in South Africa is crucial for informing cancer prevention efforts. The most recent studies of national trends in HPV-related cancer incidence in South Africa analysed data collected up to 2012.^7,8^ These studies reported large racial disparities in HPV-related cancer burden, and substantial increases in vulvar cancer (especially for younger ages) and anal cancer (especially for females),^7,8^ suggesting the epidemiology of these cancers and the prevalence of key risk factors in South Africa might be changing.

To improve understanding of HPV-related cancers in South Africa and guide policy decisions on HPV vaccination and cancer screening, continued monitoring of trends is needed. We aimed to describe the incidence of cervical, vulvar, vaginal, penile, oropharyngeal, and anal cancers in South Africa between 2011 and 2021 by sex, calendar year, age group, and race. We also aimed to evaluate trends in oral cancers (including oral cavity and oropharyngeal), stratified by anatomic subsites that tend to be HPV-related versus tobacco-related and alcohol-related.

## Methods

### Data sources

For this cross-sectional study, we used de-identified data on all incident HPV-related cancers between Jan 1, 2011, and Dec 31, 2021, from the South African National Cancer Registry of the National Health Laboratory Services,^9,10^ including sex (female or male), race (Asian, Black, Coloured, or White), age at diagnosis (≥15 years), year of diagnosis, cancer site, and cancer histology. The term “Coloured” is used by the South African Census (Statistics South Africa), which includes people with both African and Caucasian ancestry. Since 1986, the National Cancer Registry has collected all laboratory diagnosed cancers from private and public health-care laboratories in South Africa by extracting demographic and tumour data from pathology reports.^9^ In 2011, Regulation 380 of the National Health Act of 2003 made cancer a reportable disease in South Africa;^11^ thus, cancer incidence reporting after this period is considered robust and comprehensive. We used the race categories as per Statistics South Africa (the national statistics service). These specific classifications allow for measurement of historical socioeconomic disparities. In the National Cancer Registry, race information was obtained from pathology reports, which classified race through patient self-reporting.

### Outcomes

The primary outcomes were cancers with a known association with persistent HPV infection. Because HPV infects the squamous epithelium,^12^ HPV-related cancers are mostly associated with squamous cell carcinomas, with the exception of cervical cancer, of which HPV causes nearly all cases, regardless of histology. To capture cancers with the highest HPV-attributable fraction, we limited the analyses to all cervical carcinomas, vulvar squamous cell carcinomas, vaginal squamous cell carcinomas, penile squamous cell carcinomas, oropharyngeal squamous cell carcinomas, and anal squamous cell carcinomas, identified by International Classification of Diseases codes (appendix p 2). For our secondary analysis, we examined potentially HPV-related and HPV-unrelated oral and oropharyngeal squamous cell carcinomas, grouped by anatomic subsite.^13^ HPV-related sites include the base of tongue, lingual tonsil, tonsil, oropharynx, and Waldeyer’s ring. HPV-unrelated sites (mostly associated with tobacco and alcohol use) include the soft palate, uvula, oral tongue, gum, floor of mouth, hard palate, and other or unspecified parts of the mouth.^13^

### Statistical analysis

Among females and males, we described the population distribution between 2011 and 2021 overall and by race, age group, and year. Missing race was imputed using a hot-deck imputation method based on an algorithm derived from a database of approximately 1·4 million surnames and their known races.^14^ To estimate person-time (ie, denominators to calculate rates), mid-year national population estimates were obtained from Statistics South Africa, stratified by sex, year, age, and race. For each cancer type, we presented the number of cancer cases (burden), median age at diagnosis, and incidence per 100 000 person-years with corresponding 95% CIs by sex, year, age group, and race. Incidence was calculated by dividing the number of events (from the South African National Cancer Registry) by the estimated person-time (from Statistics South Africa) for all strata. All incidences were age-standardised to the 1960 Segi world standard population.^15^ To examine racial differences, we calculated incidence rate ratios (IRRs) of each cancer type for Black, Coloured, and Asian individuals compared with White individuals overall and across age groups (15–44, 45–54, 55–74, and ≥75 years). We selected White race as the reference to highlight the elevated cancer risk for Black individuals. For cancers that affect both sexes (anal squamous cell carcinoma and oropharyngeal squamous cell carcinoma), we calculated male-to-female IRRs overall and by age group. Age-standardised incidence and IRRs were calculated using SEER*Stat software (version 8.4.3.).

Temporal trends were examined by determining the best fit log-linear model and calculating the average annual percent change (AAPC) in incidence for each cancer type among females and males. Since there is etiological heterogeneity in vulvar squamous cell carcinoma by age, with most HPV-associated vulvar cancers presenting at younger ages (ie, <55 years), we also calculated trends in vulvar squamous cell carcinoma by age group. The empirical quantile method was used for all models, which were fit using weighted Bayesian Information Criterion with grid search and uncorrelated errors. Joinpoint regression was used to identify significant changes in trends at specific inflection years using a Monte Carlo Permutation method.^16^ For our secondary analysis, we calculated AAPCs in the incidence of potentially HPV-related and HPV-unrelated oral squamous cell carcinoma by sex. Pairwise tests were conducted using the parametric method to examine differences in trends between potentially HPV-related and HPV-unrelated oral squamous cell carcinomas. All trend analyses were conducted using Joinpoint software (version 5.0.2). Statistical significance was evaluated at an α level of 0·05.

### Role of the funding source

The funders of the study had no role in the study design, data collection, data analysis, data interpretation, or writing of the report.

## Results

**Figure 1:**
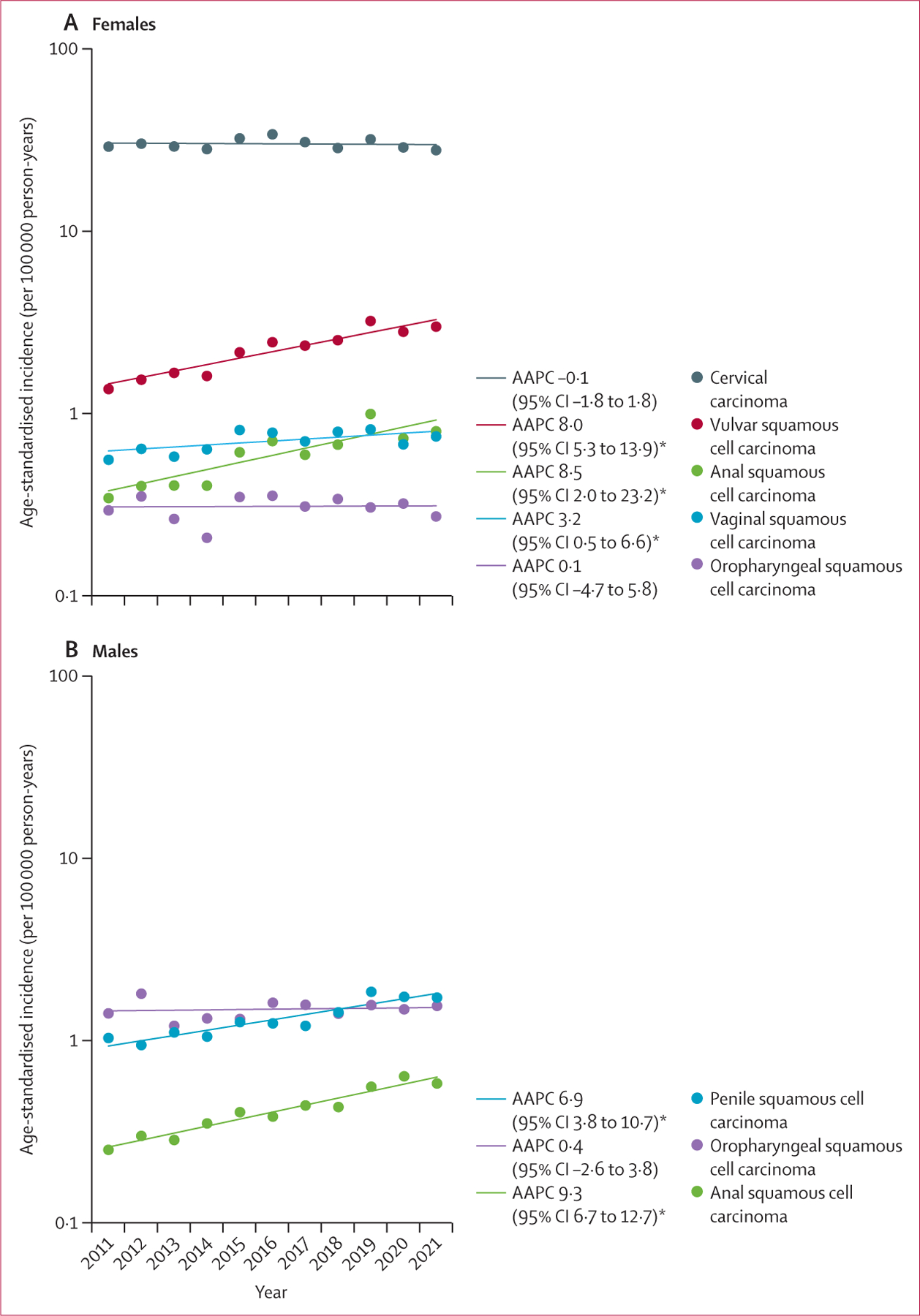
Changes in age-standardised incidence of HPV-related cancers in South Africa in women (A) and men (B) between 2011 and 2021 Joinpoints were detected for cervical carcinoma (2016), vulvar squamous cell carcinoma (2019), vaginal squamous cell carcinoma (2015), and anal squamous cell carcinoma in females only (2019); thus, the AAPC for these cancer sites reflects the AAPC of both period segments before and after the joinpoint. AAPC=average annual percentage change. HPV=human papillomavirus. *p<0·05.

**Figure 2:**
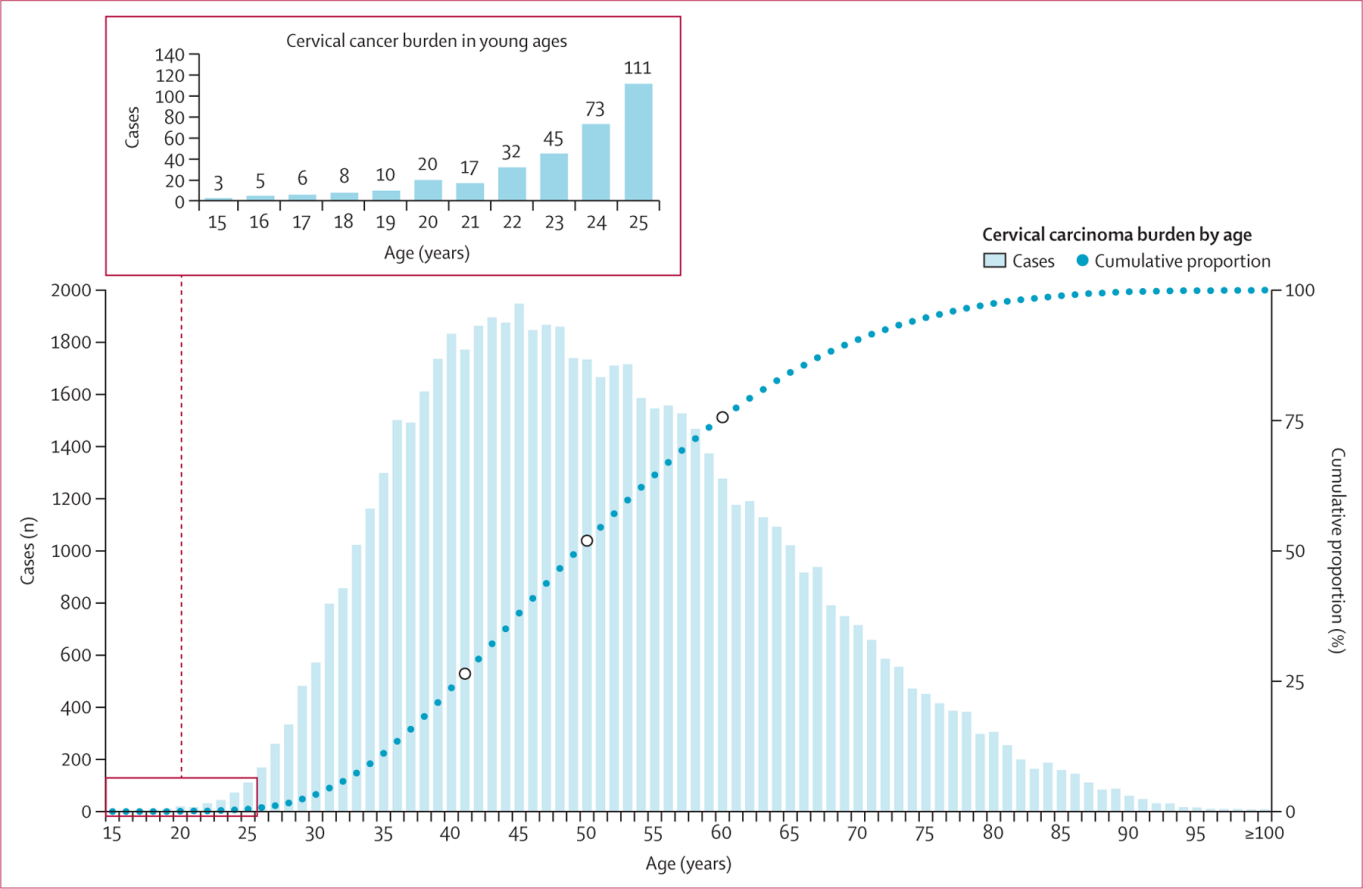
Cervical carcinoma burden among female individuals in South Africa (2011–21) by age The white dots indicate the 25th, 50th, and 75th percentiles.

Between Jan 1, 2011, and Dec 31, 2021, females aged 15 years and older in South Africa contributed to more than 227 million person-years (appendix p 3). 177 934 300 (78·1%) of 227 740 787 females were Black, 22 672 279 (10·0%) were White, 20 778 800 (9·1%) were Coloured, and 6,355,408 (2·8%) were Asian. The highest incidence of HPV-related cancers in females was cervical carcinoma (incidence 30·4 cases per 100 000 person-years [95% CI 30·2 to 30·6]; table 1). The overall trend in cervical carcinoma incidence was not significant between 2011 and 2021 (AAPC −0·1 [95% CI −1·8 to 1·8]; figure 1); incidence was stable between 2011 and 2016, after which a significant decline of 2·7% on average per year (95% CI 0·2 to 10·8) was observed between 2016 and 2021 (appendix p 4). 219 cases of cervical cancer were reported in young females aged 15–24 years (52 cases in females aged 15–20 years and 167 cases in females aged 21–24 years) between 2011 and 2021 (figure 2). Cervical carcinoma incidence was highest among females aged 55–64 years (58·5 cases per 100 000 person-years [95% CI 57·5 to 59·5]). By race, cervical carcinoma incidence was highest among Black females (35·2 cases per 100 000 person-years [34·9 to 35·5]) and lowest among Asian females (10·5 cases per 100 000 person-years [9·8 to 11·3]). Cervical carcinoma incidence among Black females was 1·8 (95% CI 1·7 to 1·8) times higher than the incidence among White females; this relative difference was highest in women aged ≥75 years (IRR for Black *vs* White females 6·9 [95% CI 6·1 to 7·9]; appendix p 5).

**Figure 3:**
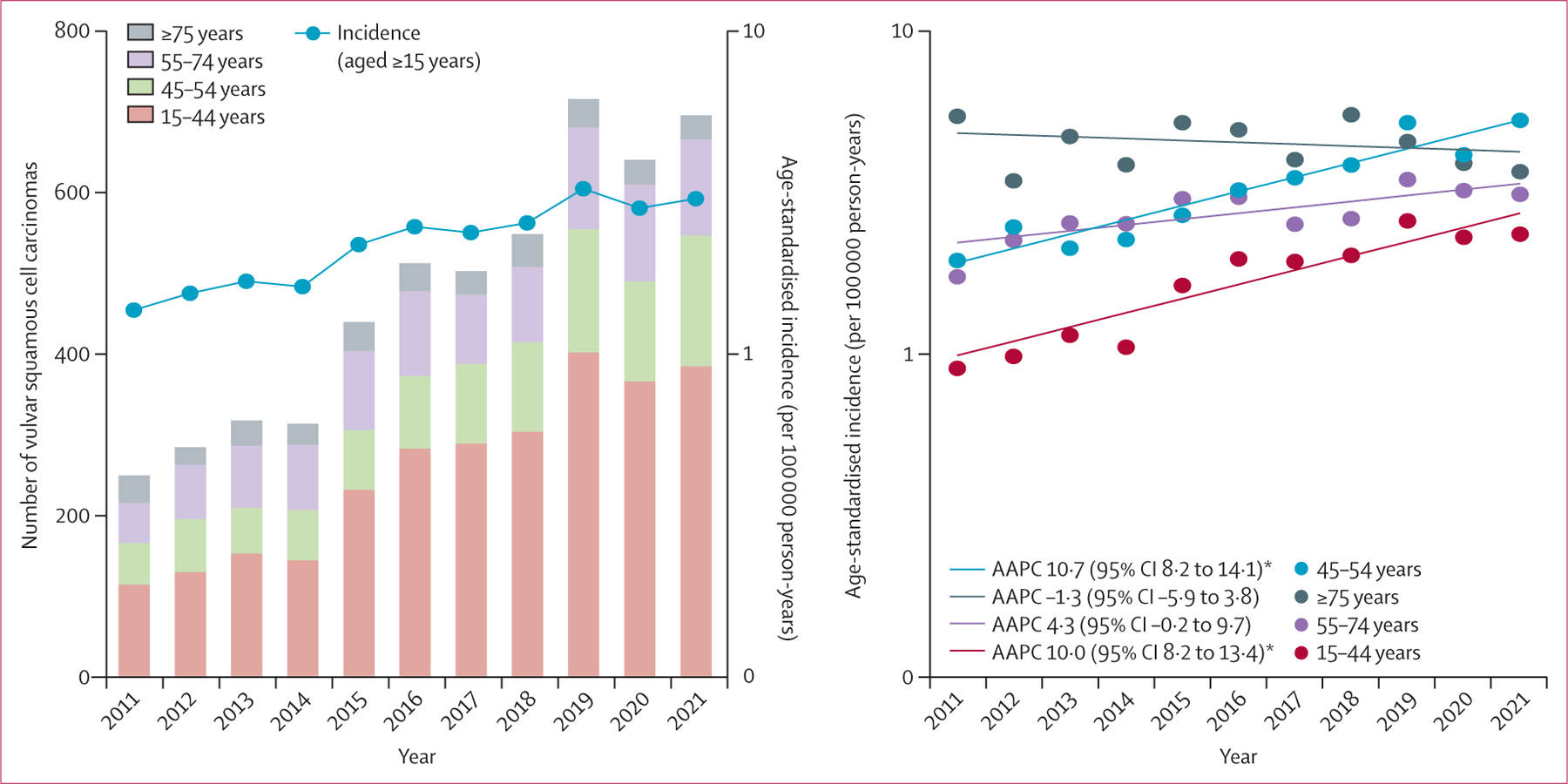
Burden, incidence, and trends in vulvar squamous cell carcinoma in South Africa (2011–21) by age group AAPC=average annual percentage change. *p<0·05.

The HPV-related cancer with the second highest incidence among females was vulvar squamous cell carcinoma (2·3 cases per 100 000 person-years [95% CI 2·2–2·4]; table 1). Vulvar squamous cell carcinoma incidence was highest in females aged 35–44 years (4·4 cases per 100 000 person-years [4·2–4·6]). Between 2011 and 2021, vulvar squamous cell carcinoma incidence increased by 8·0% (95% CI 5·3–13·9) annually (figure 1). Increases were largely driven by increases in females aged 15–54 years (AAPC for age 15–44 years 10·0% [8·2–13·4]; AAPC for age 45–54 years 10·7% [8·2–14·1]; figure 3). The incidence of vulvar squamous cell carcinoma was highest among Black females (2·3 cases per 100 000 person-years [95% CI 2·3–2·4]), which was 1·2 times (95% CI 1·1–1·3) higher than the incidence among White females (table 1; appendix p 5). The incidence of vulvar squamous cell carcinoma was higher for Black females than White females aged 15–44 years (IRR 2·4 [95% CI 2·0–3·0]), but the pattern was reversed for women aged 55 years or older (IRR for age 55–74 years 0·6 [0·5–0·7]; IRR for age ≥75 years 0·4 [0·3–0·5]).

The incidence of vaginal squamous cell carcinoma (0·7 cases per 100 000 person-years [95% CI 0·7 to 0·7]), female anal squamous cell carcinoma (0·6 cases per 100 000 person-years [0·6 to 0·7]), and female oropharyngeal squamous cell carcinoma (0·3 cases per 100 000 person-years [0·3 to 0·3]) was substantially lower than the incidence of cervical carcinoma and vulvar squamous cell carcinoma (tables 1, 2). Between 2011 and 2021, the incidence of vaginal squamous cell carcinoma increased significantly on average by 3·2% per year (95% CI 0·5 to 6·6) and the incidence of female anal squamous cell carcinoma also increased significantly on average by 8·5% per year (2·0 to 23·2). The temporal trend in the incidence of female oropharyngeal squamous cell carcinoma was not significant (AAPC 0·1 [95% CI −4·7 to 5·8]; figure 1). The incidence of vaginal squamous cell carcinoma was highest among Black females (0·8 cases per 100 000 person-years [95% CI 0·7 to 0·8]); the incidence of female anal squamous cell carcinoma was highest among Black females (0·6 cases per 100 000 person-years [0·6 to 0·6]) and White females (0·6 cases per 100 000 person-years [0·6 to 0·8]); and the incidence of female oropharyngeal squamous cell carcinoma was highest among White females (0·7 cases per 100 000 person-years [0·6 to 0·8]) and Coloured females (0·7 cases per 100 000 person-years [0·6 to 0·8]; table 2).

Between 2011 and 2021, males aged 15 years and older contributed to more than 210 million person-years (appendix p 3). 163 531 534 (77·8%) of 210 117 751 men were Black, 20 994 586 (10·0%) were White, 19 079 629 (9·1%) were Coloured, and 6 512 002 (3·1%) were Asian. The incidence of penile squamous cell carcinoma was 1·4 cases per 100 000 person-years (95% CI 1·3–1·4; table 1), and between 2011 and 2021, the incidence increased significantly (average increase 6·9% per year [95% CI 3·8–10·7]; figure 1). Incidence of penile squamous cell carcinoma was highest in men aged 75 years and older (3·9 cases per 100 000 person-years [95% CI 3·4–4·6]; table 1). The incidence of penile squamous cell carcinoma was the highest for Black males (1·5 cases per 100 000 person-years [95% CI 1·4–1·5]), which was 1·4 times (95% CI 1·2–1·6) higher than the incidence among White males; this relative difference was only observed for males aged 15–54 years (appendix p 5).

The incidence of anal squamous cell carcinoma among males was 0·4 cases per 100 000 person-years (95% CI 0·4–0·5; table 2), and between 2011 and 2021, significantly increased (average increase 9·3% per year [95% CI 6·7–12·7]; figure 1). Male anal squamous cell carcinoma incidence did not substantially differ by race overall; however, for men aged 55 years and older, the incidence was significantly lower in Black males than White males (IRR for age 55–74 years 0·5 [95% CI 0·4–0·7]; IRR for age ≥75 years 0·4 [0·1–0·8]; appendix p 5).

The incidence of oropharyngeal squamous cell carcinoma among males was 1·5 cases per 100 000 person-years (95% CI 1·4 to 1·5; table 2). The temporal trend in oropharyngeal squamous cell carcinoma incidence was not significant (AAPC 0·4 [95% CI −2·6 to 3·8]; figure 1). Oropharyngeal squamous cell carcinoma incidence was highest in men aged 55–64 years (5·4 cases per 100 000 person-years [95% CI 5·1 to 5·8]) and 65–74 years (5·3 cases per 100 000 person-years [4·9 to 5·8]; table 2). The incidence of oropharyngeal squamous cell carcinoma was higher for White men (2·4 cases per 100 000 person-years [2·2 to 2·6]) and Coloured men (2·6 cases per 100 000 person-years [2·3 to 2·9]) than Asian males (0·9 cases per 100 000 person-years [0·7 to 1·2]) and Black males (1·0 case per 100 000 person-years [1·0 to 1·1]).

The incidence of anal squamous cell carcinoma was significantly lower among males than females (IRR 0·7 [95% CI 0·6–0·8]). However, this relative difference was only observed among people aged 15–44 years (IRR for age 15–34 years 0·3 [0·2–0·4]; IRR for age 35–44 years 0·4 [0·4–0·5]), whereas no difference was identified by sex in people aged older than 45 years (appendix p 6). By contrast, the incidence of oropharyngeal squamous cell carcinoma among males was 4·8 times (95% CI 4·4–5·3) higher than the incidence among females; this relative difference generally increased with increasing age, with the largest male-to-female IRR identified among people aged 55 years and older.

When comparing the incidence of oral squamous cell carcinomas between potentially HPV-related versus HPV-unrelated cases, the incidence was much higher for HPV-unrelated oral squamous cell carcinomas than HPV-related cases, and incidence was higher in males than females (appendix p 7). Among males, patterns differed between HPV-related and HPV-unrelated oral squamous cell carcinoma incidence (pairwise p=0·001): the incidence of HPV-related oral squamous cell carcinoma was stable (AAPC 0·4% [95% CI −2·0 to 2·9]) but significant declines in HPV-unrelated oral squamous cell carcinoma (−3·3% [−4·5 to −2·0]) were observed between 2011 and 2021. Among females, trends in HPV-related and HPV-unrelated oral squamous cell carcinoma incidence were not significant and did not differ between each other (pairwise p=0·06).

## Discussion

In this national pathology-based registry study in South Africa, between 2011 and 2021, the incidence of anogenital cancer increased rapidly by 3·2% to 9·3% annually for vulvar, vaginal, and anal squamous cell carcinoma among females and for penile and anal squamous cell carcinoma among males. The overall incidence of cervical carcinoma in females and oropharyngeal squamous cell carcinoma incidence in both sexes remained stable over time; however, cervical carcinoma incidence began declining in 2016 by 2·7% per year. The incidence of cervical carcinoma was high, particularly among females aged 55–64 years (incidence of 58·5 cases per 100 000 person-years). The highest incidence of most HPV-related cancers was observed in Black South Africans with the exception of oropharyngeal squamous cell carcinoma, which was highest among White and Coloured individuals, and for anal squamous cell carcinoma, which was similar across racial groups.

Our results reflect growing concern regarding HPV-related cancers in South Africa. A previous study in South Africa reported significant increases in vulvar squamous cell carcinoma among females (16·1% annually) between 1994 and 2012 and anal squamous cell carcinoma among females (7·2% annually) and males (6·6% annually) between 2006 and 2012.^9^ Between 2011 and 2021, the incidence of both cancers continued to rise and they were the two most rapidly increasing HPV-related cancers (≥8% annually for both). Increases in vulvar squamous cell carcinoma incidence were most prominent among younger females, while trends among older females remained stable, which is likely to reflect the epidemiological heterogeneity between the two biological pathways of vulvar cancer: (1) HPV-dependent vulvar cancers, which are mostly associated with younger age and HIV coinfection; and (2) HPV-independent vulvar cancers, which are mostly associated with older age.^17,18^ Increases in vulvar cancer detection among young and middle-aged females (aged 15–54 years) could be due in part to improvements in cervical cancer screening among people with HIV after the 2017 provisions by the South African National Department of Health, which allowed all females older than 30 years in the public sector to receive three free cervical cancer screenings every 10 years.^19^ We also observed large racial differences among females for both vulvar squamous cell carcinoma and anal squamous cell carcinoma incidence by age group. The incidence of vulvar squamous cell carcinoma and anal squamous cell carcinoma was more than two times higher in Black females aged 15–44 years than White females, but these differences inverted for older ages, among which Black females had significantly lower incidence compared with White females.

Heterogeneity by race and age are potentially rooted in the negative consequences of Apartheid, during which spatial segregation, economic disenfranchisement, and systemic racism were prominent, which have led to persistent disparities in HIV prevalence, access to health-care services, such as antiretroviral therapy, and risk factors associated with HPV acquisition and progression, especially for Black South Africans.^20,21^ Due to these systemic barriers and disparities, in addition to sexual network structures, prolonged conjugal separation, legacy of migrant labour for Black men, and the cultural value systems of polygamy in African culture versus European culture, the prevalence of HPV-HIV coinfection is disproportionately high in young Black females in South Africa, resulting in more rapid progression to invasive cancer.^21,22^ Furthermore, differences might be perpetuated by early sexual debut among Black South African girls, who, due to structural inequalities and poverty, are more likely to have transactional relationships, and in many cases, unequal power dynamics with male counterparts, leading to increased risk of unsafe sex and high HIV prevalence among this population.^23^ Due to historical and structural factors, race is a proxy for socioeconomic status, HIV prevalence, and access to health-care in South Africa; therefore, our race-stratified results could be impacted by unmeasured confounders, such as social drivers.

In the previous two decades, patterns in the incidence of penile and vaginal squamous cell carcinoma in South Africa have shifted. Between 1994 and 2012, the incidence of penile cancer in South Africa significantly declined by 3% annually;^8^ however, our study found that between 2011 and 2021, the incidence significantly increased by 7% annually. Additionally, the incidence of vaginal squamous cell carcinoma was stable between 1994 and 2012,^8^ but significantly increased by 3% annually between 2011 and 2021. Changes in trends could partly be explained by enhanced case ascertainment after the South African National Health Act made cancer a mandatory reportable disease in 2011.^11^ However, other epidemiological reasons for the upward trends should be further investigated.

Despite cervical cancer screening availability, cervical carcinoma incidence remains high and largely unchanged, with some declines starting in 2016. Although free cervical cancer screenings are available, only 56% of South African females aged 30–49 years were ever screened for cervical cancer in 2020.^24^ Common barriers to screening include lack of access and knowledge of cervical cancer.^25^ Even among screened females, some never receive results and few book follow-up appointments; even when appointments are booked, waiting times for care could be up to 15 months while the cancer freely progresses.^26^ South African females with confirmed high-grade cervical lesions have low treatment rates (as low as 16%),^27^ which emphasises the inefficiencies within the health-care system in linking women with confirmed precancers to appropriate and timely treatment. A possible solution to the loss in follow-up could be offering same-day so-called screen and treat using HPV-based screening. Although declines in cervical cancer from 2016 onward are promising, cervical cancer screening, timely treatment of precancers, and HPV vaccination should continue to be prioritised to accelerate declines.

The impact of HPV vaccination on cancer reduction in South Africa is not expected to occur for at least a decade, when HPV-vaccinated girls reach cancer-susceptible ages. The free school-based HPV vaccination programme in South Africa was only introduced among girls aged 9–10 years in 2014.^22^ HPV vaccination coverage was initially high (>80% in 2014) but substantially declined during the COVID-19 pandemic.^22^ In addition to interruptions in HPV vaccination programmes during the COVID-19 pandemic, HPV vaccine supply shortages remain a major limitation. As a solution, South Africa implemented a single-dose HPV vaccination programme in April, 2024, which could enhance coverage, reduce costs, and simplify vaccine delivery moving forward. Importantly, South African males are relying on herd immunity and reduced HPV prevalence in females to reduce HPV-related cancers in males; however, the prevention of HPV cancers that are more prevalent in males who have sex with males, such as anal cancer, should also be prioritised.

Our study also showed significant declines in HPV-unrelated oral squamous cell carcinomas among males between 2011 and 2021. This could be due to the strict tobacco control laws implemented in 1999, which banned public smoking, tobacco advertisements and sponsorships, and the sale of tobacco products to minors.^28^ Our results also showed that HPV-unrelated oral squamous cell carcinoma incidence was much higher among males than females, likely due to higher tobacco use among South African males (26%) than females (13%).^28^ Despite the promising declines in HPV-unrelated oral squamous cell carcinoma incidence among males, the smoking rate among South Africans remains the highest in sub-Saharan-Africa, signalling the need for continued tobacco control for cancer prevention.^28^

Our study was limited by the lack of tumour confirmation for HPV positivity. To accurately characterise trends in HPV-driven cancers, evaluating markers of HPV-induced carcinogenesis in tumours is warranted, especially for non-cervical cancers. Additionally, we did not have data on HIV or antiretroviral therapy status; thus, findings might have masked important differences between people with and without HIV. For example, incidence of HPV-related cancers is significantly higher among people with HIV than those without HIV, and this association is higher among younger people than older people.^29^ To better understand the role of HIV in HPV-related cancers, future studies should characterise trends in anogenital and oropharyngeal cancers disaggregated by HIV and antiretroviral therapy status. Because the registry data were obtained from pathology reports, non-laboratory diagnosed cancers were not included, such as clinically diagnosed cancers (around 14–27%),^30^ for which a cervical cancer was visible on the cervix but a biopsy was not taken for histological confirmation; therefore, our results might be underestimated.

A key strength of our study was the access to national-level pathology report data from all public and private health-care laboratories in the country, which report to the South African National Cancer Registry, the country’s main source of cancer incidence data that is used to generate reports of annual cancer statistics that inform cancer policy and guidelines. Consequently, our data had large sample sizes with more robust estimates than other studies that only included one or two provinces or regions in South Africa. Our study is also the most recent and comprehensive study of trends in HPV-related cancers in South Africa.

In conclusion, we observed increasing trends in most HPV-related cancers in South Africa between 2011 and 2021. Cervical cancer contributed to most HPV-related cancers; therefore, strengthening the cervical cancer cascade of care from primary and secondary prevention to treatment is crucial to reduce the overall burden of HPV-related cancers in South Africa. Furthermore, we identified the emergence and increasing importance of vulvar cancers among young females. High incidence of cervical and vulvar cancer indicates that females bear the brunt of HPV-related cancers; integrating HPV-related cancer prevention into sexual and reproductive programmes or through decentralised mechanisms, such as using home-based self-sampling, could be strategic. Because cancer services are mostly centralised in high-income urban areas, which limits access in low-income and rural areas, decentralising cancer care services and building cancer care capacity in primary health-care settings throughout the country would minimise cancer disparities. Additionally, considering that Black individuals represent nearly 80% of the South African population, have the highest incidence of most HPV-related cancers, and have the highest HIV burden in the country, reducing HPV-HIV coinfections in this population by increasing access to and uptake of HIV prevention and treatment methods could accelerate reductions in HPV-related cancer incidence. Health promotion efforts in South Africa could also be improved by better equipping front-line health-care workers with knowledge about preventing HPV-HIV coinfections.

## Supplementary Material

MMC1

## Figures and Tables

**Table 1:** Age-standardised incidence per 100 000 person-years of sex-specific HPV-related cancers in the South African National Cancer Registry (2011–21)

	Cervical carcinoma			Vulvar squamous cell carcinoma			Vaginal squamous cell carcinoma			Penile squamous cell carcinoma		
	n	Incidence (95% CI)*	Median age, years (IQR)	n	Incidence (95% CI)*	Median age, years (IQR)	n	Incidence (95% CI)*	Median age, years (IQR)	n	Incidence (95% CI)*	Median age, years (IQR)
Overall	65 176	30·4 (30·2, 30·6)	50 (41–60)	5247	2·3 (2·2–2·4)	43 (36–55)	1525	0·7 (0·7–0·7)	51 (41–62)	2280	1·4 (1·3–1·4)	50 (43–61)
Race												
Black	54 640	35·2 (34·9–35·5)	50 (41–60)	4093	2·3 (2·3–2·4)	41 (35–49)	1190	0·8 (0·7–0·8)	49·5 (40–60)	1745	1·5 (1·4–1·5)	49 (42–57)
White	4993	19·8 (19·2–20·4)	49 (41–59)	638	1·9 (1·8–2·1)	63 (49–76)	162	0·5 (0·4–0·6)	60 (50–70)	312	1·0 (0·9–1·2)	63 (52–74)
Coloured†	4149	19·5 (18·9–20·1)	51 (42–60)	337	1·6 (1·4–1·8)	52 (41–65)	110	0·5 (0·4–0·6)	56·5 (47–66)	146	0·9 (0·8–1·1)	58·5 (46–67)
Asian	753	10·5 (9·8–11·3)	52 (42–63)	109	1·4 (1·2–1·7)	65 (51–74)	39	0·5 (0·4–0·7)	61 (52–72)	54	0·9 (0·7–1·1)	57 (49–66)
Unknown	641	..	49 (41–59)	70	..	45·5 (37–59)	24	..	57·5 (45–67)	23	..	52 (44–63)
Age group, years												
15–24	219	0·4 (0·3–0·4)	..	53	0·1 (0·1–0·1)	..	11	0·0 (0·0–0·0)	..	5	0·0 (0·0–0·0)	..
25–34	5770	9·6 (9·4–9·9)	..	937	1·6 (1·5–1·7)	..	139	0·2 (0·2–0·3)	..	119	0·2 (0·2–0·2)	..
35–44	16 878	41·1 (40·5–41·7)	..	1836	4·4 (4·2–4·6)	..	343	0·8 (0·7–0·9)	..	551	1·4 (1·3–1·5)	..
45–54	17 675	57·2 (56·3–58·0)	..	1049	3·4 (3·2–3·6)	..	388	1·3 (1·1–1·4)	..	737	2·8 (2·6–3·0)	..
55–64	13 340	58·5 (57·5–59·5)	..	630	2·8 (2·5–3·0)	..	318	1·4 (1·3–1·6)	..	427	2·5 (2·3–2·8)	..
65–74	7405	53·4 (52·2–54·6)	..	392	2·8 (2·5–3·1)	..	210	1·5 (1·3–1·7)	..	279	3·0 (2·7–3·4)	..
≥75	3889	49·7 (48·2–51·3)	..	350	4·5 (4·0–5·0)	..	116	1·5 (1·2–1·8)	..	162	3·9 (3·4–4·6)	..
Year												
2011–12	10 487	30·0 (29·4–30·6)	50 (41–61)	539	1·5 (1·3–1·6)	46 (35–59)	208	0·6 (0·5–0·7)	52 (42–61)	273	1·0 (0·9–1·1)	51 (40–63)
2013–14	10 646	29·0 (28·4–29·6)	50 (41–61)	636	1·6 (1·5–1·8)	46 (36–60)	226	0·6 (0·5–0·7)	50·5 (40–61)	313	1·1 (1·0–1·2)	49 (41–61)
2015–16	12 884	33·5 (33·0–34·1)	50 (41–60)	957	2·3 (2·2–2·5)	43 (36–57)	310	0·8 (0·7–0·9)	53 (40–65)	374	1·3 (1·1–1·4)	51 (43–63)
2017–18	12 126	30·0 (29·5–30·6)	49 (41–60)	1056	2·5 (2·3–2·6)	43 (36–53·5)	301	0·8 (0·7–0·8)	51 (42–62)	422	1·3 (1·2–1·5)	50 (43–60)
2019–21	19 033	29·8 (29·4–30·2)	49 (41–60)	2059	3·0 (2·9–3·2)	43 (37–52)	480	0·7 (0·7–0·8)	51 (41–63)	898	1·8 (1·7–1·9)	51 (45–60)

HPV=human papillomavirus.

* Per 100 000 person-years.

† The term Coloured is used by the South African Census (Statistics South Africa), which includes people with both African and Caucasian ancestry.

**Table 2:** Age-standardised incidence per 100 000 person-years of oropharyngeal squamous cell carcinoma and anal squamous cell carcinoma in the South African National Cancer Registry by sex (2011–21)

	Anal squamous cell carcinoma						Oropharyngeal squamous cell carcinoma					
	Female			Male			Female			Male		
	n	Incidence (95% CI)*	Median age, years (IQR)	n	Incidence (95% CI)*	Median age, years (IQR)	n	Incidence (95% CI)*	Median age, years (IQR)	n	Incidence (95% CI)*	Median age, years (IQR))
Overall	1396	0·6 (0·6–0·7)	44 (37–56·5)	755	0·4 (0·4–0·5)	49 (41–58)	614	0·3 (0·3–0·3)	60 (52–67)	2200	1·5 (1·4–1·5)	–
Race												
Black	1039	0·6 (0·6–0·6)	41 (36–50)	511	0·4 (0·4–0·4)	46 (39–53)	200	0·1 (0·1–0·2)	57 (50–65)	979	1·0 (1·0–1·1)	59 (53–65)
White	207	0·6 (0·6–0·8)	62 (50–71)	136	0·5 (0·4–0·6)	61 (51–70)	228	0·7 (0·6–0·8)	62 (55–67)	692	2·4 (2·2–2·6)	61 (55–68)
Coloured†	95	0·4 (0·4–0·5)	52 (40–65)	72	0·4 (0·3–0·5)	53·5 (47·5–62)	138	0·7 (0·6–0·8)	59 (52–66)	425	2·6 (2·4–2·9)	59 (53–64)
Asian	17	0·2 (0·1–0·4)	49 (44–58)	20	0·3 (0·2–0·5)	57 (50·5–67·5)	18	0·2 (0·1–0·4)	62 (48–67)	55	0·9 (0·7–1·2)	59 (54–66)
Unknown	38	..	47 (39–59)	16	..	50 (40·5–62)	30	..	58 (51–69)	49	..	61 (53–64)
Age group, years												
15–34	234	0·2 (0·2–0·2)	..	71	0·1 (0·0–0·1)	..	11	0·0 (0·0–0·0)	..	21	0·0 (0·0–0·0)	..
35–44	481	1·2 (1·1–1·3)	..	198	0·5 (0·4–0·6)	..	42	0·1 (0·1–0·1)	..	115	0·3 (0·2–0·4)	..
45–54	293	1·0 (0·8–1·1)	..	234	0·9 (0·8–1·0)	..	148	0·5 (0·4–0·6)	..	513	1·9 (1·8–2·1)	..
55–64	180	0·8 (0·7–0·9)	..	144	0·8 (0·7–1·0)	..	216	1·0 (0·8–1·1)	..	914	5·4 (5·1–5·8)	..
65–74	125	0·9 (0·7–1·1)	..	76	0·8 (0·6–1·0)	..	154	1·1 (0·9–1·3)	..	498	5·3 (4·9–5·8)	..
≥75	83	1·1 (0·8–1·3)	..	32	0·8 (0·5–1·1)	..	43	0·6 (0·4–0·7)	..	139	3·4 (2·8–4·0)	..
Year												
2011–12	139	0·4 (0·3–0·4)	47 (35–63)	75	0·3 (0·2–0·3)	51 (43–62)	105	0·3 (0·3–0·4)	60 (53–68)	396	1·6 (1·5–1·8)	58 (52–65)
2013–14	153	0·4 (0·3–0·5)	49 (36–60)	92	0·3 (0·3–0·4)	51·5 (42·5–61·5)	80	0·2 (0·2–0·3)	58 (51–63·5)	318	1·3 (1·1–1·4)	59 (53–65)
2015–16	268	0·7 (0·6–0·7)	44 (36–57)	127	0·4 (0·3–0·5)	46 (40–56)	127	0·4 (0·3–0·4)	60 (51–67)	388	1·5 (1·3–1·6)	60 (53–65)
2017–18	267	0·6 (0·6–0·7)	44 (38–56)	142	0·4 (0·4–0·5)	50 (43–59)	123	0·3 (0·3–0·4)	60 (54–67)	416	1·5 (1·3–1·6)	60 (54–66)
2019–21	569	0·8 (0·8–0·9)	43 (37–55)	319	0·6 (0·5–0·7)	49 (41–57)	179	0·3 (0·3–0·3)	60 (52–67)	682	1·5 (1·4–1·7)	60 (54–67)

* Per 100 000 person-years.

† The term Coloured is used by the South African Census (Statistics South Africa), which includes people with both African and Caucasian ancestry.

## Research in context

### Evidence before this study

We searched PubMed for peer-reviewed articles published between Jan 1, 2014, and Oct 8, 2024 using the search terms: (“South Africa”[Title/Abstract] AND incidence[Title/Abstract] AND trend*[Title/Abstract]) AND (cervical[Title/Abstract] OR vulvar[Title/Abstract] OR vaginal[Title/Abstract] OR penile[Title/Abstract] OR oropharyngeal[Title/Abstract] OR anal[Title/Abstract]). Our search yielded ten articles. Four articles were not applicable because they did not report on human papillomavirus (HPV)-related cancer incidence or trends over time. Of the remaining six studies, three reported only on one or two provinces or regions in South Africa and an additional study reported on cervical cancer trends among people with HIV only, which might not be generalisable to the entire country. Only two studies described national trends in HPV-related cancer incidence in South Africa, of which one study reported that annual cervical cancer incidence declined by 1% between 1994 and 2009, but increased significantly (by 3%) annually for females aged 25–34 years between 2000 and 2009. The other study reported significant increases in vulvar and penile cancer incidence between 1994 and 2012, but stable trends in vaginal cancer incidence during this time. The study also reported increases in anal cancer incidence between 2006 and 2012 and decreases in oropharyngeal cancer between 1996 and 2005.

### Added value of this study

To our knowledge, our study is the most up-to-date and comprehensive evaluation of national HPV-related cancer trends, including cervical, vulvar, vaginal, penile, oropharyngeal, and anal cancers, in South Africa. Using the South African National Cancer Registry, we reported trends in HPV-related cancers between 2011 and 2021 and compared rates by sex, age, and race. Our results add to the existing literature by demonstrating the persistently high burden and incidence of cervical cancer in South Africa, particularly among Black females (incidence 35·2 cases per 100 000 person-years), and among females aged 55–64 years (58·5 cases per 100 000 person-years). We also observed a continual and rapid rise in vulvar and anal cancer incidence and observed a shift in trends for penile cancer (from significant declines to significant increases), vaginal cancer (from stable trends to significant increases), and oropharyngeal cancer (from significant declines to stable trends) from previous reports. A major finding was that the incidence of vulvar cancer rapidly increased by 10% among women aged 15–44 years and by 11% among women aged 45–54 years on average per year between 2011 and 2021, which is notable considering that vulvar cancers among these age groups are most often associated with HPV–HIV co-infection.

### Implications of all the available evidence

In the past decade, the incidence of anogenital cancers has significantly increased in South Africa, with the exception of cervical cancer, which generally remained stable but started declining in 2016. Despite these declines, cervical cancer remains a large problem in South Africa, emphasising the need to improve cervical cancer prevention, including prioritising HPV vaccination, cervical cancer screening and follow-up, and timely treatment of cervical precancers. Additionally, rapid increases in vulvar cancer incidence among young females highlights the increasing importance of HPV and HIV prevention in South Africa.
