# Supplementary material for: Changes in incidence of HPV-related cancers in South Africa (2011–21): a cross-sectional analysis of the South African National Cancer Registry
Source: Lancet Glob Health. Author manuscript; Available in PMC 2025 Aug 20. (PMC12366449; doi:10.1016/S2214-109X(25)00065-8)
Supplement: MMC1 [file NIHMS2086508-supplement-MMC1.pdf]

THE LANCET
Global Health

Supplementary appendix

This appendix formed part of the original submission and has been peer reviewed. We post it as supplied by the authors.

Supplement to: Shing JZ, Mashele S, Tsegaye AT, et al. Changes in incidence of HPV-related cancers in South Africa (2011–21): a cross-sectional analysis of the South African National Cancer Registry. *Lancet Glob Health* 2025; **13:** e1101–1110.

Supplementary Appendix

Contents **Page**

1. Supplementary Table 1. Site and histology codes by cancer type……………………………………………...2
2. Supplementary Table 2. Population estimates in South Africa among female and male individuals aged 15 years and older, 2011-2021……………………………………………………………………………………...3
3. Supplementary Table 3. Detected inflection years and annual percent changes in sex-specific HPV-related cancers in the South African National Cancer Registry, 2011-2021……………………………………………...4
4. Supplementary Figure 1. Age-standardized incidence rate ratios of HPV-related cancers among female and male individuals in South Africa (2011-2021) by race across age groups……………………………………………...5
5. Supplementary Figure 2. Age-adjusted incidence rates of anal squamous cell carcinoma and oropharyngeal squamous cell carcinoma in South Africa (2011-2021) by age group and sex…………………………………...6
6. Supplementary Figure 3. Trends in potentially HPV-related and HPV-unrelated oral squamous cell carcinomas in South Africa (2011-2021) by sex………………………………………………………………………………7

Supplementary Table 1. Site and histology codes by cancer type.

Cancer Type	Sex	Site Codes	Histology Codes
**HPV-Related Cancers**			
Cervical Carcinoma	Female	C53·0, C53·1, C53·8, C53·9	8010-8671, 8940, 8941
Vulvar Squamous Cell Carcinoma	Female	C51·0, C51·1, C51·2, C51·8, C51·9	8050-8076, 8078, 8083, 8084, 8094
Vaginal Squamous Cell Carcinoma	Female	C52·9	8050-8076, 8078, 8083, 8084, 8094
Penile Squamous Cell Carcinoma	Male	C60·0, C60·1, C60·2, C60·8, C60·9	8050-8076, 8078, 8083, 8084, 8094
Anal Squamous Cell Carcinoma	Female, Male	C21·0, C21·1, C21·2, C21·8	8050-8076, 8078, 8083, 8084, 8094
Oropharyngeal Squamous Cell Carcinoma	Female, Male	• Base of tongue: C01·9<br>• Lingual tonsil: C02·4<br>• Tonsil: C09·0, C09·1, C09·8, C09·9<br>• Oropharynx: C10·0, C10·1, C10·2, C10·3, C10·4, C10·8, C10·9<br>• Waldeyer’s ring: C14·2	8050-8076, 8078, 8083, 8084, 8094
**Oral (Oral Cavity and Oropharyngeal) Squamous Cell Carcinoma**			
HPV-Related	Female, Male	Oropharyngeal<br>• Base of tongue: C01·9<br>• Lingual tonsil: C02·4<br>• Tonsil: C09·0, C09·1, C09·8, C09·9<br>• Oropharynx: C10·0, C10·1, C10·2, C10·3, C10·4, C10·8, C10·9<br>• Waldeyer’s ring: C14·2	8050-8076, 8078, 8083, 8084, 8094
HPV-Unrelated	Female, Male	Oropharyngeal<br>• Soft palate: C05·1<br>• Uvula: C05·2<br>Oral Cavity<br>• Oral Tongue: C02·0, C02·1, C02·3, C02·5, C02·8, C02·9<br>• Gum: C03·0, C03·1, C03·9<br>• Floor of mouth: C04·0, C04·1, C04·8, C04·9<br>• Hard Palate: C05·0, C05·8, C05·9<br>• Other/unspecified parts of the mouth: C06·0, C06·1, C06·2, C06·8, C06·9	8050-8076, 8078, 8083, 8084, 8094

Supplementary Table 2. Population estimates in South Africa among female and male individuals aged 15 years and older, 2011-2021.

	Overall	Female	Male
	n (%)	n (%)	n (%)
Total (N)	437,858,538	227,740,787	210,117,751
Race			
Black	341,465,834 (78·0%)	177,934,300 (78·1%)	163,531,534 (77·8%)
White	43,666,865 (10·0%)	22,672,279 (10·0%)	20,994,586 (10·0%)
Coloured [a]	39,858,429 (9·1%)	20,778,800 (9·1%)	19,079,629 (9·1%)
Asian	12,867,410 (2·9%)	6,355,408 (2·8%)	6,512,002 (3·1%)
Age Group, years			
15-24	108,936,373 (24·9%)	54,138,947 (23·8%)	54,797,426 (26·1%)
25-34	113,968,579 (26·0%)	56,457,528 (24·8%)	57,511,051 (27·4%)
35-44	82,362,104 (18·8%)	41,759,196 (18·3%)	40,602,908 (19·3%)
45-54	57,502,041 (13·1%)	30,907,213 (13·6%)	26,594,828 (12·7%)
55-64	39,952,995 (9·1%)	22,764,223 (10·0%)	17,188,772 (8·2%)
65-74	23,208,582 (5·3%)	13,895,494 (6·1%)	9,313,088 (4·4%)
75+	11,927,864 (2·7%)	7,818,186 (3·4%)	4,109,678 (2·0%)
Year			
2011-2012	73,927,655 (16·9%)	38,622,603 (17·0%)	35,305,052 (16·8%)
2013-2014	76,386,398 (17·4%)	39,827,505 (17·5%)	36,558,893 (17·4%)
2015-2016	78,910,793 (18·0%)	41,063,980 (18·0%)	37,846,813 (18·0%)
2017-2018	81,452,247 (18·6%)	42,302,459 (18·6%)	39,149,788 (18·6%)
2019-2021	127,181,445 (29·0%)	65,924,240 (28·9%)	61,257,205 (29·2%)

[a] The term "Coloured" is used by the South African Census (Statistics South Africa), which includes people with both African and Caucasian ancestry.

Supplementary Table 3. Detected inflection years and annual percent changes in sex-specific HPV-related cancers in the South African National Cancer Registry, 2011-2021.

Cancer	Inflection Year	Period	Annual Percent Change (95% CI)
**Female**			
Cervical Carcinoma	2016	2011-2016	2·5 (-0·1, 14·1)
		2016-2021	-2·7 (-10·8, -0·2)*
Vulvar Squamous Cell Carcinoma			
All ages	2019	2011-2019	10·5 (3·3, 43·3)*
		2019-2021	-1·7 (-15·6, 12·3)
Age 15-44 years	2019	2011-2019	13·9 (11·8, 23·3)*
		2019-2021	-4·5 (-12·8, 7·9)
Age 45-54 years	--	--	--
Age 55-74 years	--	--	--
Age 75+ years	--	--	--
Vaginal Squamous Cell Carcinoma	2015	2011-2015	8·9 (3·1, 31·8)*
		2015-2021	-0·5 (-11·4, 2·7)
Anal Squamous Cell Carcinoma	2019	2011-2019	12·7 (-7·4, 113·3)
		2019-2021	-7·1 (-36·1, 22·3)
Oropharyngeal Squamous Cell Carcinoma	--	--	--
**Male**			
Penile Squamous Cell Carcinoma	--	--	--
Anal Squamous Cell Carcinoma	--	--	--
Oropharyngeal Squamous Cell Carcinoma	--	--	--

*Asterisks denote p<0·05· Dashes denote no inflection year identified.

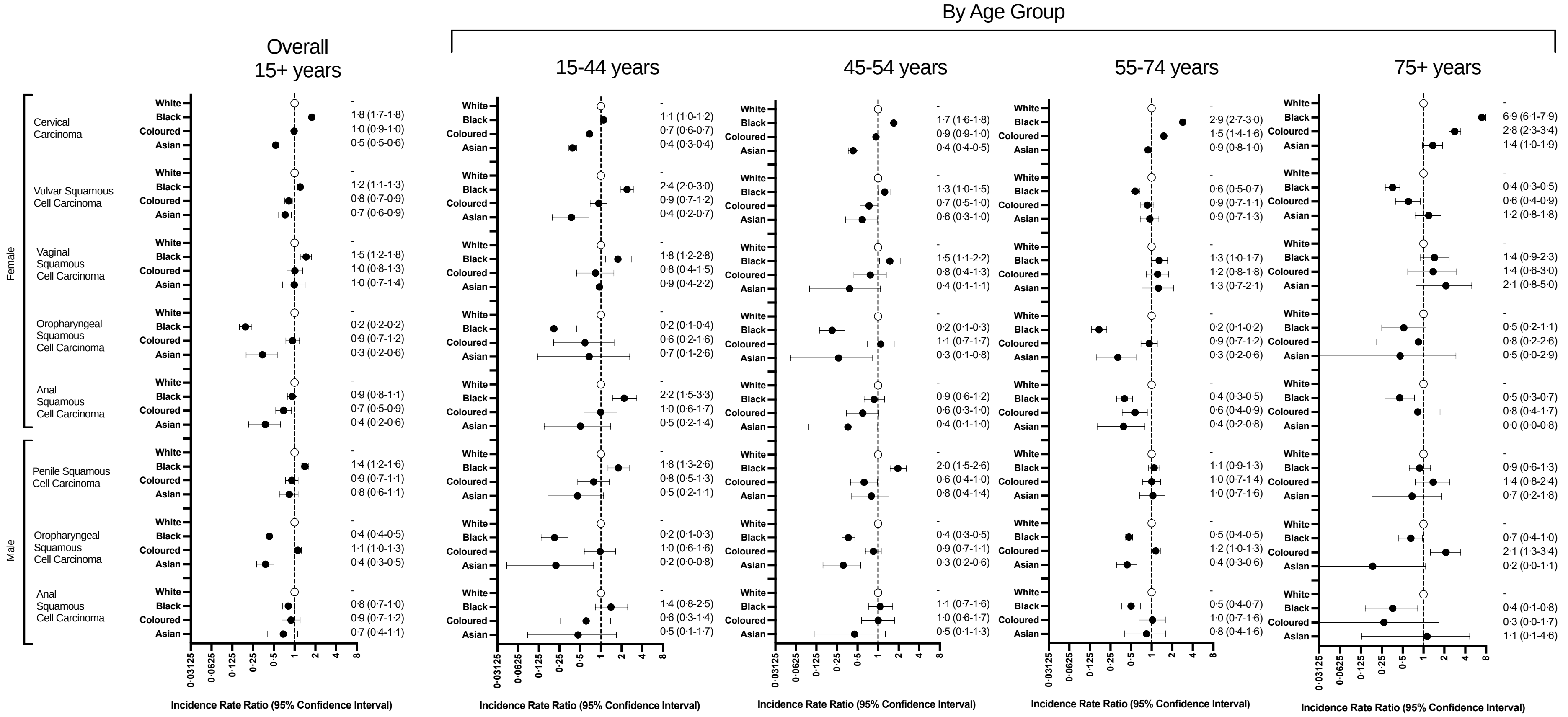

Supplementary Figure 1. Age-standardized incidence rate ratios of HPV-related cancers among female and male individuals in South Africa (2011-2021) by race across age groups.

Abbreviations: HPV = human papillomavirus. The term "Coloured" is used by the South African Census (Statistics South Africa), which includes people with both African and Caucasian ancestry. Although the Black population is the largest racial group in South Africa, we selected White race as the reference group to highlight the elevated cancer risk for Black individuals.

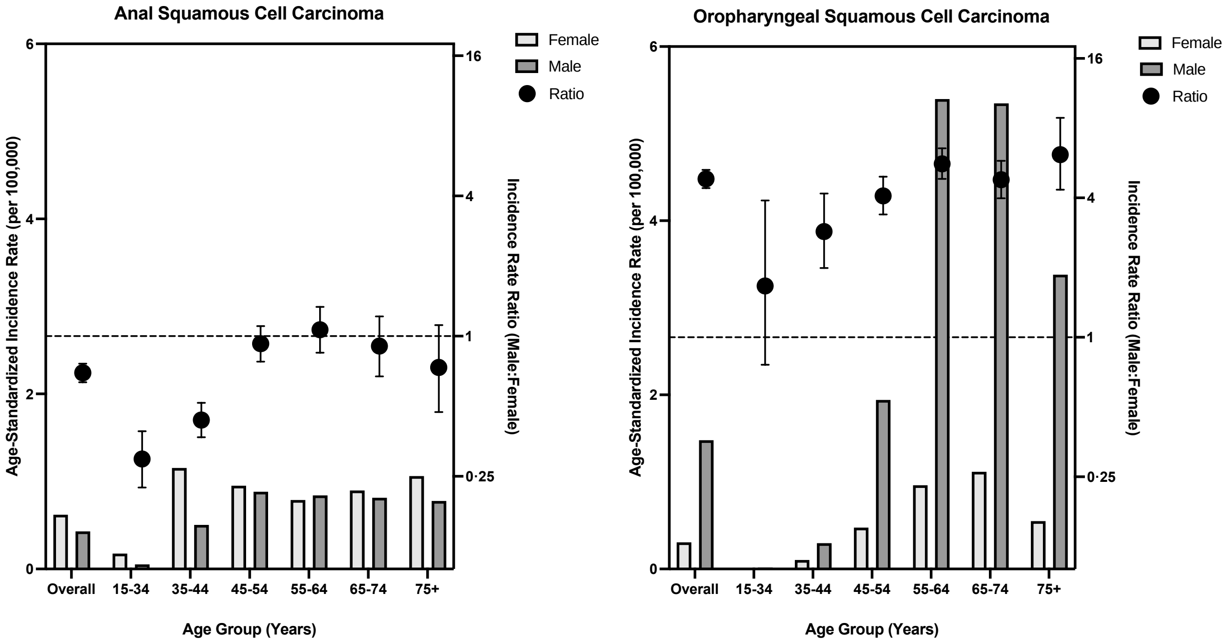

Supplementary Figure 2. Age-adjusted incidence rates of anal squamous cell carcinoma and oropharyngeal squamous cell carcinoma in South Africa (2011-2021) by age group and sex.

The dots represent the male-to-female incidence rate ratio (female as the reference group), with corresponding 95% confidence intervals. The dashed horizontal line represents a male-to-female incidence rate ratio of 1·0.

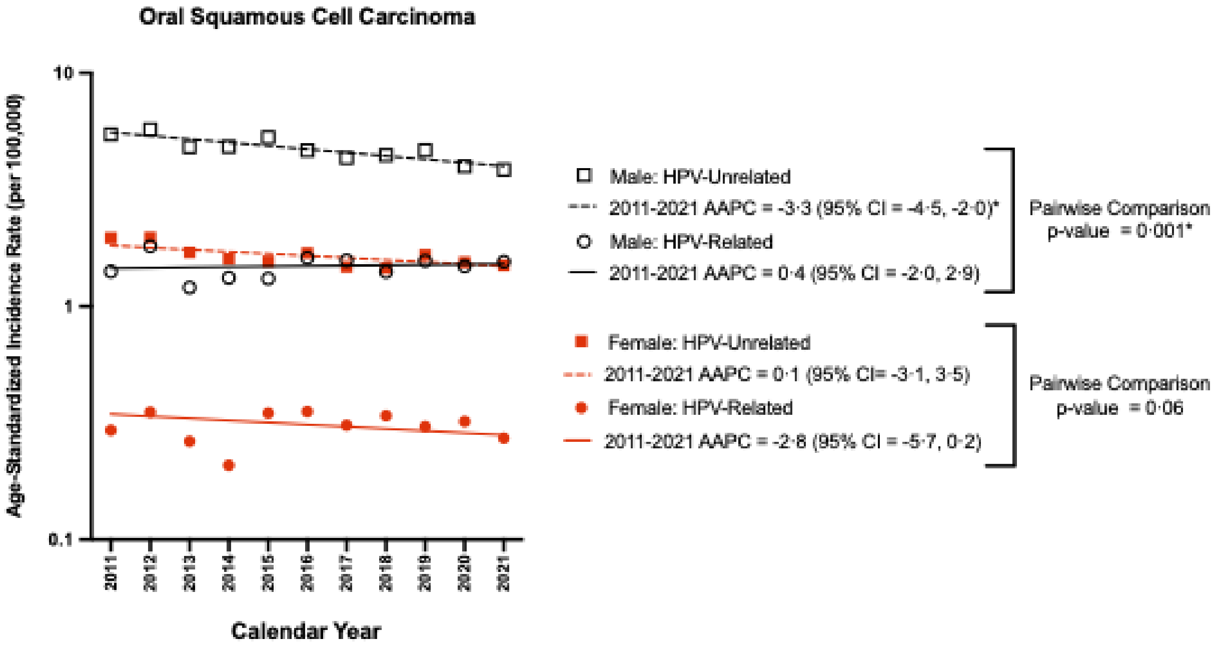

Supplementary Figure 3. Trends in potentially HPV-related and HPV-unrelated oral squamous cell carcinomas in South Africa (2011-2021) by sex.

*Asterisks denote p<0·05. HPV-related sites included base of tongue, lingual tonsil, tonsil, oropharynx, and Waldeyer's ring. HPV-unrelated sites included oral tongue, gum, floor of mouth, palate, and other/unspecified parts of the mouth. 95% confidence intervals for APCs were calculated using the parametric method to be able to compute pairwise comparison tests for parallelism. Abbreviations: AAPC = annual percent change; CI = confidence interval; HPV = human papillomavirus
